# Miltefosine in the Treatment of Cutaneous Leishmaniasis Caused by *Leishmania braziliensis* in Brazil: A Randomized and Controlled Trial

**DOI:** 10.1371/journal.pntd.0000912

**Published:** 2010-12-21

**Authors:** Paulo R. Machado, Julia Ampuero, Luiz H. Guimarães, Leonardo Villasboas, Ana T. Rocha, Albert Schriefer, Rosana S. Sousa, Anette Talhari, Gerson Penna, Edgar M. Carvalho

**Affiliations:** 1 Serviço de Imunologia, Hospital Universitário Prof. Edgard Santos, Universidade Federal da Bahia, Salvador, Brazil; 2 Núcleo de Medicina Tropical, Universidade de Brasília, Brasília, Brazil; 3 Fundação de Medicina Tropical do Amazonas, Manaus, Brazil; AP-HP, Service de Parasitologie-Mycologie, France

## Abstract

**Background:**

Cutaneous leishmaniasis (CL) is treated with parenteral drugs for decades with decreasing rate cures. Miltefosine is an oral medication with anti-leishmania activity and may increase the cure rates and improve compliance.

**Methodology/Principal Findings:**

This study is a randomized, open-label, controlled clinical trial aimed to evaluate the efficacy and safety of miltefosine versus pentavalent antimony (Sb^v^) in the treatment of patients with CL caused by *Leishmania braziliensis* in Bahia, Brazil. A total of 90 patients were enrolled in the trial; 60 were assigned to receive miltefosine and 30 to receive Sb^v^. Six months after treatment, in the intention-to-treat analyses, the definitive cure rate was 53.3% in the Sb^v^ group and 75% in the miltefosine group (difference of 21.7%, 95% CI 0.08% to 42.7%, p = 0.04). Miltefosine was more effective than Sb^v^ in the age group of 13–65 years-old compared to 2–12 years-old group (78.9% *versus* 45% p = 0.02; 68.2% *versus* 70% p = 1.0, respectively). The incidence of adverse events was similar in the Sb^v^ and miltefosine groups (76.7% vs. 78.3%). Vomiting (41.7%), nausea (40%), and abdominal pain (23.3%) were significantly more frequent in the miltefosine group while arthralgias (20.7%), mialgias (20.7%) and fever (23.3%) were significantly more frequent in the Sb^v^ group.

**Conclusions:**

This study demonstrates that miltefosine therapy is more effective than standard Sb^v^ and safe for the treatment of CL caused by *Leishmania braziliensis* in Bahia, Brazil.

**Trial Registration:**

Clinicaltrials.gov Identifier NCT00600548

## Author Summary

Cutaneous leishmaniasis (CL) is characterized by skin ulcerations and occurs in rural poor areas of developing countries. It is treated with daily injections of antimony for 20 days, which is associated with irregular use and increasingly lower cure rates. Miltefosine is an oral medication with activity against the agent of CL (*Leishmania*). We have studied the efficacy and safety of miltefosine compared with antimony in patients with CL caused by *Leishmania braziliensis* in Bahia, Brazil. A total of 90 patients participated; 60 received miltefosine and 30 were treated with antimony. Six months after treatment, 75% of patients treated with miltefosine were cured, compared with 53% of the patients in the antimony group, a difference considered significant (p = 0.04). We also found that miltefosine was more effective than antimony in adults than in children. The incidence of side effects was similar with both drugs (76.7% vs. 78.3%), but all patients were able to finish the treatments. Our study shows that miltefosine is more effective than antimony for the treatment of CL in Bahia, Brazil and can contribute to the control of this disease due to its activity and easier administration.

## Introduction

The principal species causing cutaneous leishmaniasis (CL) in Brazil is *Leishmania braziliensis* which most often leads to a cutaneous form of the disease characterized by one or more ulcers with raised borders, most frequently located on the upper and lower extremities, but also found on the head, face, and trunk [1], [2]. Although CL is a self-limited disease, approximately 3 to 5% of subjects infected with *L. braziliensis* will eventually develop mucosal disease or disseminated leishmaniasis, both considered severe forms of leishmaniasis [2]–[4].

Pentavalent antimony (Sb^v^) by intramuscular or intravenous route remains the first-line drug for the treatment of CL, a therapy that is moderately toxic and difficult to administer in poor rural areas. In an endemic area of *L. braziliensis* transmission in Bahia, Brazil, cure rates after Sb^v^ therapy are becoming increasingly lower and vary from 50% to 90% [5]–[7]. Factors contributing to this variability are not yet fully understood. The development of parasite resistance [8] to a drug used for decades and irregular adherence by the patients due to the daily schedule of parenteral route during 20 days, could be main factors in determining the increasingly failure rate to Sb^v^ [5]–[7]. Other alternative drugs like pentamidine and amphothericin B are also of parenteral use and the former may require hospitalization.

Miltefosine, a phosphatidylcholine analogue, is an active antileishmanial oral drug used for the treatment of visceral leishmaniasis in India [9]. The clinical efficacy of miltefosine for New World CL was investigated in trials conducted in Central and South America. The cure rates varied from one leishmania species to another, with *L. panamensis* having 82% of cure rate, *L. mexicana* 60% and *L braziliensis* 33% respectively [10]. More recently in CL caused by *L. braziliensis* in patients from Bolivia, Soto et al reported a 88% cure rate [11]. These data shows that CL cure rate upon miltefosine treatment varies among Leishmania species and also between the same species from different endemic areas. Accordingly, it is demonstrated that different treatment outcome after antimony therapy is found in CL caused by *L. braziliensis* from different endemic regions in Brazil [5], [6], [12]. There is no data about the use of miltefosine in CL caused by *L. braziliensis* in Brazil, where this species is considered the most aggressive and prevalent agent of the disease [13].

This randomized and controlled trial evaluated the efficacy and safety of miltefosine versus meglumine antimoniate (Sb^v^) for the treatment of CL in a *L. braziliensis* endemic rural area in Bahia, Brazil.

## Methods

### Ethics Statement

This trial has been conducted according to the principles expressed in the Declaration of Helsinki. Prior to enrollment in the study, all patients received a written copy of study policy which was reviewed with them individually by an independent party. A written informed consent was obtained for all adult patients, and from parents or guardians of minors. This study was approved by the Ethics Committee of the Federal University of Bahia, in Salvador, Brazil (CEP/MCO/UFBA-Par/Res 034/2007).

### Endemic area

All subjects were recruited at the health post of Corte de Pedra, located 260 km southeast of Salvador, the capital of Bahia, Brazil. This clinic is a referral center for the diagnosis and treatment of CL, with an average of 1,000 new cases per year. *L. braziliensis* has been the unique causal agent identified in this area in the last 15 years [14].

### Patient selection

The criteria used for the diagnosis of CL were the presence of a typical ulcerated lesion and a positive Montenegro intradermal skin test in a subject living in the endemic area. A typical CL ulcerated lesion is characterized by a round shape and raised borders associated with regional adenopathy. This classical clinical picture together with a positive leishmania skin test is highly specific for CL in the endemic area [2], [5]. Patients were then selected based on the following inclusion criteria: 1) age between 2 and 65 years; 2) a maximum of 5 ulcers with no more than 2 body regions involved; 3) lesion size between 10 and 50 mm in a single dimension; and 4) a period of less than 90 days from the onset of the first ulcer. All subjects were submitted to a punch biopsy to obtain material for leishmania culture and PCR. Patients with a prior history of CL or antimony use, patients with evidence of mucosal or disseminated disease, pregnant or breastfeeding mothers, and patients with HIV or any systemic severe disease were excluded. A total of 90 patients were enrolled in the study.

### Group Assignment

A randomization table was obtained with Statacorp LP 9, Texas USA. Group assignments were made after assessment that patient had met all eligibility criteria and no patients were withdrawn after randomization because of ineligibility. The 90 patients were randomly assigned at a rate of 2∶1 allocation to receive miltefosine for 28 days or Sb^v^ for 20 days in two age groups (2–12 years-old and 13–65 years-old).

### Intradermal skin test

The Leishmania antigen used for intradermal skin testing was obtained from *Leishmania amazonensis* strain (MHOM-BR-86BA-125). The volar surface of the left forearm was injected with 25 µg of antigen in 0.1 mL of distilled water, and the largest diameter of induration was measured at 48–72 hours. The test was considered positive for induration greater than 5 mm.

### Parasitology

#### Parasite Culture

Needle aspiration of a skin lesion was performed, and aspirates were cultured in Nicolle-McNeal-Novy medium overlaid with modified liver infusion triptase medium. Cultures were kept at 25°C and examined twice weekly. Isolates were characterized by use of a panel of monoclonal antibodies.

### PCR for leishmania DNA

DNA isolation was carried out from biopsy samples using the Wizard Genomic DNA purification kit (Promega Corporation – USA). Purified DNA was resuspended in TE buffer and stored frozen at −20°C until use. Detection of the subgenus Viannia applied the primers 5′-GGGGTTGGTGTAATATAGTGG-3′ and 5′-CTAATTGTGCACG-3′. For Leishmania genus detection we have used the primers 5′-(G/C)(G/C)(C/G)CC(A/C)CTAT(A/T)TTA CAC CAA CCC C-3′ and 5′-GGG GAG GGG CGT-3′. Amplification mixes consisted of 25 pmol each primer; 1.2 mM MgCl_2_; 0.2 mM dNTP; 2.5 U Taq DNA polymerase; 10× PCR buffer; 2 uL of target DNA. Amplifications were carried out in a Veriti 96-well thermal cycler (Applied Biosystems – USA). Viannia detection applied 35 cycles of 1 minute at 94°C, 1 minute at 60°C and 1 minute at 72°C. Amplicons were fractionated in 1.3% agarose gels, stained with ethidium bromide and photographed under UV light using a UVP Vision Works LS apparatus (UVP – USA). The *Leishmania* specific band consists of 120 base pairs, and that for Viannia of 750 base pairs.

### Drug administration

All study volunteers were treated as outpatients. Miltefosine (Impavido, Zentaris GmbH) was supplied in blisters containing 10 mg or 50 mg capsules. Meglumine antimoniate (Glucantime, Aventis) was supplied in vials of 5 ml containing 81 mg/Sb^v^/ml. Miltefosine was administered orally at the total target daily dosage of 2.5 mg/kg of body weight (maximum daily dose of 150 mg) for 28 consecutive days. Daily dose was divided in two or three intakes, given always with meals according to the following weight scale: patients with ≥15 kg and ≤29 kg - total dose of 50 mg/day; patients with ≥30 kg and ≤45 kg - total dose of 100 mg/day; patients with ≥46 kg - total dose of 150 mg/day. Meglumine antimoniate (Sb^v^) was administered intravenously at a dose of 20 mg Sb^v^/kg/day for 20 consecutive days (maximum daily dose of 3 ampoules or 1215 mg/Sb^v^). At every weekly visit patients returned the blisters for verification of regular use and adherence. Sb^v^ was administered daily in health posts near from patient's home. In these cases the administration's date and dosage were registered and signed by the health care provider.

All women in child bearing age were submitted to beta HCG test to exclude pregnancy. The use of a parenteral contraceptive during and for 2 months after treatment was done in all women in child bearing age.

### Study Procedures

Complete hemogram, aminotransferases (AST, ALT), alkaline phosphatase, potassium, sodium, urea, creatinine, and urine chemistry were determined in all patients on day -1, weekly thereafter up to the end of therapy, and after 15 and 30 days of the end of therapy. Those with abnormal parameters were followed-up until normalization. At each weekly return for drug dispensation patients were monitored for adverse events (AE). Bidirectional measurements of ulcers were taken of the patients' lesions at the initial visit, and at each follow-up visit with standardized caliper. The area involved was calculated as the product of the two measurements. A standardized digital photograph was also taken from each patient's lesions at the same time points.

Patients were seen for follow-up at 2 weeks, 1, 2, 4 and 6 months post-therapy. In the event that a patient did not return for follow-up at the specified time, visits were conducted in the patient's home in the same day or within 7 days of the missed appointment.

#### Clinical endpoints criteria

Primary end point: cure at 6 months after the end of therapy. Secondary end-points: a) cure at 2 months after the end of therapy; b) data from clinical and laboratorial adverse events. All lesions were also categorized as either active or healed (cured) at follow-up visits. Only lesions with complete re-epithelialization, without raised borders, infiltrations or crusts were considered healed. Evaluation of the lesions was performed by 2 clinicians (LHG, EMC) who were unaware of the group assignment of all patients.

Clinical and laboratory AE were graded according to the Common Terminology Criteria for Adverse Event v3.0 (CTCAE) of the National Cancer Institute (ctep.cancer.gov/protocolDevelopment/electronic_applications/docs/ctcaev3.pdf).

### Statistical Analysis

The results are presented as proportions, interquartile ranges (IQR), 95% confidence intervals (95% CI), means and standard-deviations (SD). The normally distributed variables were compared using the t test. The proportions were compared with the Chi-square or Fisher test when appropriate.

The sample size of 90 patients was obtained by calculating the number of participants needed for 80% power (β = 0.2) to detect an absolute difference as large as 25% in the rate of cure between the two treatment groups with a statistical significance of 5% (α = 0.05). The intention-to-treat (ITT) analysis was used to calculate the cure rates. All statistical analyses were performed with the software SPSS 9.0 for Windows. A value of p<0.05 established the level of statistical significance.

## Results

**Figure 1:**
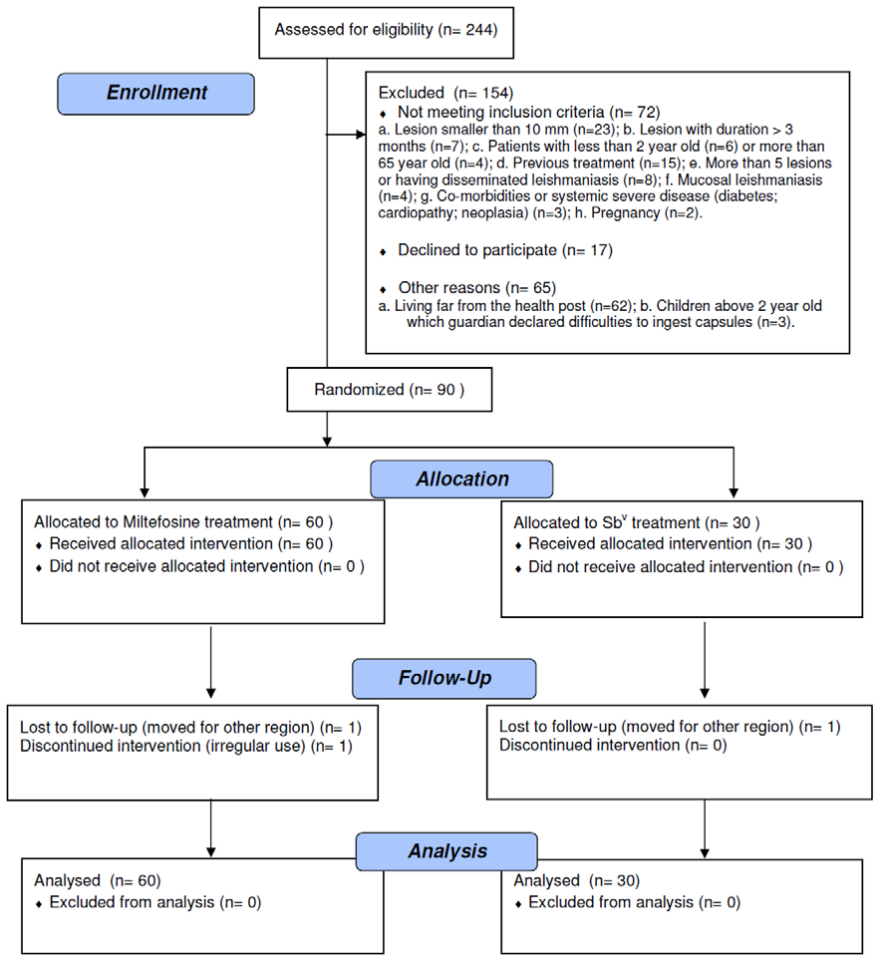
Trial flowchart.

From the 244 CL patients screened from July 2007 and August 2008, 154 subjects met the inclusion criteria, 90 were included in the trial and completed the treatment, and 87 were followed for the entire 6 months after therapy, up to March 2009 (Figure 1). The patients ranged in age from 4 to 65 years with the mean age of 22±15 years, which did not differ significantly between the two treatment groups (p = 0.85). Overall, there was a predominance of male patients (67.8% vs. 32.2%), and there were statistically more males in the miltefosine group than in the Sb^v^ group (75.0% vs. 53.3%, p = 0.04). The majority presented with a single lesion (76.7%), and significantly more patients in the miltefosine group had ≥2 lesions (30% vs. 10%, p = 0.04). The lesion with the greatest area at presentation was considered to be the main lesion. The main lesion area was not different between treatment groups (410.6 vs. 461.2 mm^2^, p = 0.47). The clinical characteristics of patients in the two treatment groups and age groups (2–12 years and 13–65 years) are shown in Table 1. A positive culture for leishmania promastigotes was obtained in 52 out of 90 patients (57.7%). *L braziliensis* was identified in 41 obtained samples from biopsy by PCR. In 26 patients (28.8%) neither PCR nor culture identified leishmania.

**Table 1:** Baseline characteristics of the 90 patients with Cutaneous Leishmaniasis included in the trial and treated with Sb^v^ or miltefosine.

Characteristics	Sb^v^(n = 30)		Miltefosine(n = 60)		P value	
	Age group in years				Age2–12 y	Age13–65 y
	2–12 y(n = 10)	13–65 y(n = 20)	2–12 y(n = 22)	13–65 y(n = 38)		
Male/female	16/14		45/15		0.04	
Number of male/female per age group	5/5	11/9	15/7	30/8	0.44	0.07
Age (years) ± SD (range)	22.7±14.7 (7–65)		22.0±15.2 (4–59)		0.85	
No. of lesions (%)1≥2	27 (90.0)3 (10.0)		42 (70.0)18 (30.0)		0.04	
No. of lesions per age group					NA	NA
1	8	19	15	27		
2	0	0	6	7		
3	1	1	0	4		
4	1	0	1	0		
5	0	0	0	0		
Area of lesion (mm^2^) ± SD	410.6		461.2		0.47	

### Efficacy

Two months after the end of the treatment, 60% (18/30) of patients in the Sb^v^ group had cured lesions, compared with 81.7% (49/60) in the miltefosine group. The cure rates at 6 months of follow-up were 53.3% (16/30) in the Sb^v^ group and 75% (45/60) in the miltefosine group (p = 0.04) (Table 2). The absolute difference was 21.7% (95% CI 0.08% to 42.7%). The mean time to cure was 75±15 days in the Sb^v^ group and 73±17 days in the miltefosine group (p = 0.63).

**Table 2:** Follow-up endpoints results in patients with CL treated with Sb^v^or miltefosine.

Follow-up endpoints	Sb^v^	Miltefosine	P value
	N = 30	N = 60	
**End of Treatment**	**20 days**	**28 days**	
Apparent cure (%)	1 (3.3)	1 (1.7)	0.61
Failure (%)	29 (96.7)	59 (98.3)	
Lost in follow-up (%)	0	0	
**2 months after treatment**			
Apparent cure (%)	18 (60.0)	49 (81.7)	0.03
Failure (%)	12 (40.0)	11 (18.3)	
Lost in follow-up (%)	1	2	
**6 months after treatment**			
Definitive cure (%)	16 (53.3)	45 (75.0)	0.04
Clinical Failure (%)	14 (46.7)	15 (25.0)	
Lost in follow-up (%)	1	2	
95% CI of cure rate	35.5% to 71.2%	64.0% to 86.0%	
Difference in cure rate and 95% CI	21.7% (0.08 to 42.7)		

In age groups 2–12 years-old and 13–65 years-old cure rates at 6 months for miltefosine and Sb^v^ were 68.2% (15/22) *versus* 70% (7/10) (p = 1.0), and 78.9% (30/38) *versus* 45% (9/20) (p = 0.02), respectively.

Two patients (one from miltefosine group and the other from Sb^v^ group) were lost for follow-up after the end of the treatment. One patient in the miltefosine group was excluded by irregular use of the medication.

Relapses occurred in both groups (two patients treated with Sb^v^ and four patients treated with miltefosine) between the 2 months and 6 months after the end of the treatment. In four patients (miltefosine  = 3 and Sb^v^ = 1) there was a reactivation of the ulcer and the two others (miltefosine  = 1 and Sb^v^ = 1) had reactivation of the infiltration in the borders of the healed ulcer.

To make sure that the absence of leishmania identification did not influence the outcome, we compared the 6 month cure rate for patients with negative culture or PCR versus those with positive culture or PCR. In the miltefosine group, the cure rate of patients with positive culture or PCR (n = 41) was 75.6% compared to 73.7% in the patients with negative culture or PCR (n = 19). In the Sb^v^ group, patients with positive culture or PCR (n = 23) had 47.8% of cure rate compared to 71.4% in the patients with negative culture or PCR (n = 7). However, the number of Sb^v^ patients without parasitological confirmation is very low to allow any conclusion. Indeed if we exclude all parasitological negative patients from the analysis of the primary outcome, the cure rate in the miltefosine group (75.6%) is higher as compared to Sb^v^ group (47.8%).

### Toxicity and Tolerability

The frequency of AE was similar in the Sb^v^ and miltefosine groups (76.7% vs. 78.3%, p = 0.86) but they were reported more commonly in patients ≥13 years-old than those <13 years-old (88.3% vs. 66.7%, p = 0.07). Although the detection of AE was similar, the type of side effects varied widely between the treatment arms (Table 3). The AE that were significantly more frequent in the miltefosine group were vomiting (41.7%), nausea (40%) and abdominal pain (23.3%). In the Sb^v^ group, arthralgias (20.7%), mialgias (20.7%) and fever (23.3%) were significantly more frequent than in the miltefosine group (Table 3). Others common AE were diarrhea (10% of miltefosine patients) and headache (43% of Sb^v^ patients). None of the reported AE required complete discontinuation of therapy in any patient. In the miltefosine group, one patient presented CTC grade 3 vomiting (6 episodes in 24 hrs but did not require IV fluids) and other patient grade 3 diarrhea (increase of ≥7 stools per day over baseline and interfering with activities of daily living, but did not require hospitalization). Both were able to continue the treatment with miltefosine after a period of 3 to 5 days of interruption and oral fluid supplementation. One patient in the Sb^v^ group presented one episode of CTC grade 3 urticaria at the end of therapy. This patient needed to use oral antihistaminic for 7 days with total regression of urticaria.

**Table 3:** Clinical Toxicity Data in patients with CL treated with Sb^v^ or miltefosine.

Side effect symptoms (%)	Sb^v^(N = 30)	CTC Grade	Miltefosine(N = 60)	CTC Grade	P value**
Vomiting	1 (3.3)	2	25 (41.7)	1 (17)*2 (7)3 (1)	0.0001
Nausea	3 (10.0)	1	24 (40.0)	1 (18)2 (6)	0.003
Abdominal pain	0 (0)	_	14 (23.3)	1 (14)	0.004
Diarrhea	1 (3.3)	1	6 (10.3)	1 (4)2 (1)3 (1)	0.42
Epigastralgia	2 (6.7)	1	5 (8.6)	1 (5)	1.0
Headache	13 (43.3)	1 (9)2 (4)	17 (28.3)	1 (15)2 (2)	0.17
Dizziness	4 (13.3)	1 (3)2 (1)	8 (13.3)	1 (7)2 (1)	1.0
Arthralgias	6 (20.7)	1 (3)2 (3)	0 (0)	_	0.001
Mialgias	6 (20.7)	1 (3)2 (3)	0 (0)	_	0.001
Fever	7 (23.3)	1 (5)2 (2)	2 (3.3)	1 (2)	0.006
Anorexia	5 (16.7)	1	7 (11.7)	1 (7)	0.5
Urticaria	3 (10.0)	1 (2)3 (1)	1 (1.7)	2 (1)	0.1

*In parenthesis, the number absolute of patients for each CTC grade.

**P was obtained comparing the incidence of each symptoms between miltefosine and Sb^v^ groups.

Mild and transient raised liver enzymes was detected in less than 5% of patients in both groups. No raised levels of urea and creatinine or sodium and potassium abnormalities were detected (data not shown).

## Discussion

The treatment of CL caused by *L. braziliensis* with Sb^v^ in rural endemic areas in Brazil has been associated with decreasing cure rates, in a setting where the parenteral route can be implicated with unsatisfactory adherence. Additionally, the monotherapy of CL with Sb^v^ after several decades may induce Leishmania resistance. It also has been shown that CL caused by *L. braziliensis* has higher therapeutic failure when compared to CL due to other Leishmania species [15]. Therefore the development of new therapeutic strategies associated to a better patient compliance and higher efficacy is needed to a better control of CL.

Our study is the first to evaluate the efficacy of miltefosine in CL caused by *L. braziliensis* in adults and children in Brazil. Our data shows that a better therapeutic outcome is found after miltefosine treatment with 75% of cure, compared to 53% in the Sb^v^ group irrespective of the age group. While we found no difference between Sb^v^ (70%) and miltefosine (68%) cure rates in children, we observed that in patients above 12 years-old the superiority of miltefosine is higher, with 79% of cure compared to 45% with Sb^v^ treatment. The reasons for miltefosine be more effective than antimony on adults and have similar efficacy in children is not completely understood. A decrease in the efficacy of miltefosine in children could be explained by the difficulty to ingest the capsules and also by differences in the biodisposition of the drug in children. However, the efficacy of miltefosine in children and adults was similar in the present study, and the difference found in cure rates of miltefosine and Sb^v^ was due to the high rate of antimony failure in the adult population. It can not be ruled out that no difference between the two drugs was documented in the children population due to the small number of children in the study, and consequently lack of power to detect the differences. Alternatively the genetic polymorphism of *L. braziliensis* could explain the decreased susceptibility to Sb^v^ in isolates infecting the adult population. We have shown that *L. braziliensis* is polymorphic in the endemic region of Corte de Pedra [14], and severe forms of the disease with lower response to antimony therapy are observed predominantly in the adult population with agricultural labor [3], [4], [16]. For instance, in disseminated leishmaniasis less than 40% of patients are cured with one Sb^v^ course [4], and atypical CL failure to Sb^v^ treatment is documented in up to 95% of the patients [16].

Although the use of miltefosine is associated with several AE (as well as Sb^v^ therapy), we reported no SAE or biochemical abnormalities that caused treatment to be abandoned. Miltefosine is a safe and effective oral treatment of CL caused by *L. braziliensis* in Bahia, Brazil and should be regarded as an option for CL therapy in rural endemic areas of *L. braziliensis* transmission in Brazil.

## Supporting Information

Checklist S1 Miltefosine consort - checklist.
(0.06 MB PDF)

Protocol S1 Miltefosine protocol.
(0.11 MB PDF)
