# Supplementary material for: Miltefosine in the Treatment of Cutaneous Leishmaniasis Caused by Leishmania braziliensis in Brazil: A Randomized and Controlled Trial
Source: PLoS Negl Trop Dis. 2010 Dec 21;4(12):e912. doi: 10.1371/journal.pntd.0000912 (PMC3006132; doi:10.1371/journal.pntd.0000912)
Supplement: Protocol S1 — Miltefosine protocol. (0.11 MB PDF) [file pntd.0000912.s002.pdf]

Study Protocol: D-18506-Z020 Version: September 2006

**Study Type**	Interventional	**Clinical phase**	II
**Study Design**	Allocation: Randomized<br>Control: Active Control<br>Endpoint Classification: Safety/Efficacy Study<br>Intervention Model: Parallel Assignment<br>Masking: Open Label<br>Primary Purpose: Treatment	**Project**	Miltefosine in Cutaneous Leishmaniasis

TITLE OF THE STUDY

Clinical trial to assess efficacy and safety of orally administered miltefosine in Brazilian patients with cutaneous leishmaniasis and with standard care as active control

Principal Investigators

Paulo R. L. Machado[1]
Edgar M. Carvalho[1]
Julia Ampuero[2]
Leonardo Villasboas[1]
Luis H. Guimarães[1]

Co-investigators

Ana T. Rocha[1]
Gerson O. Penna[2]
Anette Talhari[3]

[1]Serviço de Imunologia, Hospital Universitário Prof. Edgard Santos, Universidade Federal da Bahia, Salvador, Bahia, Brazil.
[2] Núcleo de Medicina Tropical, Universidade de Brasília, Brasília, DF, Brazil.
[3] Fundação de Medicina Tropical do Amazonas, Manaus, Amazonas, Brazil.

Study center

Corte de Pedra - Bahia
Federal University of Bahia

Sponsors and Collaborators

Conselho Nacional de Desenvolvimento Científico e Tecnológico
Ministério de Ciência e Tecnologia
Ministério da Saúde
Zentaris Gmb H

TABLE OF CONTENTS Page

**1. Background**	3
**2. Objectives**	3
**3. Methodology**	3
3.1 Study design	3
3.2 Study population	3
3.3 Description of treatments	5
3.4 Evaluation of clinical response	6
**4. Study procedures**	9
4.1 Pre-study evaluation / selection	9
4.2 Assessments during the study	9
4.3 Assessments at the end of treatment	9
4.4 Assessments 2 weeks, 1 month and 2 months after the end of the treatment	10
4.5 Assessments 6 months after the end of the treatment	10
4.6 Flowchart	10
**5. Legal aspects of bioethics, biosafety, intellectual property and other legal determination**	11
**6. References**	12

1. BACKGROUND

The principal species causing cutaneous leishmaniasis (CL) in Brazil is *Leishmania braziliensis* which most often leads to a cutaneous form of the disease characterized by one or more ulcers with raised borders. Approximately 3 to 5% of subjects infected with *L. braziliensis* will eventually develop mucosal disease or disseminated leishmaniasis, both considered severe forms of leishmaniasis.

Pentavalent antimony (Sb^v) by intramuscular or intravenous route remains the first-line drug for the treatment of CL, a therapy that is moderately toxic and difficult to administer in poor rural areas. Miltefosine, a phosphatidylcholine analogue, is an active antileishmanial oral drug used for the treatment of visceral leishmaniasis in India. The clinical efficacy of miltefosine for New World CL was investigated in trials conducted in Central and South America. The cure rates varied from one leishmania species to another, with *L. panamensis* having 82% of cure rate, *L. mexicana* 60% and *L braziliensis* 33% respectively. There is no data about the use of miltefosine in CL caused by *L. braziliensis* in Brazil, where this species is considered the most aggressive and prevalent agent of the disease.

2. OBJECTIVES

The objective of this trial is to assess if therapeutic activity and safety of oral miltefosine patients with CL caused by *L. braziliensis* in an endemic area of Bahia state is similar or superior to the standard treatment (meglumine antimoniate – Glucantime®).

<u>Primary endpoint</u>:
- Rate of final cures (6 month after treatment)

<u>Secondary endpoints</u>:
- Rate of initial cure (2 month after treatment)
- Incidence and severity of adverse events

3. METHODOLOGY

3.1 Study design

Open trial, randomized controlled phase II trial comparing miltefosine with pentavalent antimony in the treatment of cutaneous leishmaniasis.

3.2 Study population

It is planned to include in this study 90 patients, with 30 patients in Group 1 (2 to 12 years old) and 60 patients in Group 2 (13 to 65 years old). Of this total, 60 patients will receive the study drug (Miltefosine), while the remaining 30 will be part of the control group (receive Glucantime ®). Ratio miltefosine / standard : 2/1.

3.2.1 Inclusion criteria

- Newly diagnosed (untreated) cutaneous leishmaniasis with localized lesions and visualization of amastigotes in tissue samples or a positive culture or diagnosed by polymerase chain reaction (PCR) methods or by intradermal skin testing (Montenegro test)
- Number of lesions: 1 to 5 ulcerative lesions
- Lesion´s diameter: 1 to 5 cm.
- Age: **Group 1: 2 to 12** years; **Group 2: 13 to 65** years
- Sex: male and female patients eligible (no effort to be made to balance the study for gender)

3.2.2 Exclusion criteria

Safety concerns:

- Thrombocyte count $<30 \times 10^9$/l
- Leukocyte count $<1 \times 10^9$/l
- Hemoglobin <5 g/100 ml
- ASAT, ALAT, AP ≥3 times upper limit of normal range
- Bilirubin ≥2 times upper limit of normal range
- Serum creatinine or BUN ≥1.5 times upper limit of normal range
- Evidence of serious underlying disease (cardiac, renal, hepatic or pulmonary)
- Immunodeficiency or antibody to HIV
- Severe protein and/or caloric malnutrition (Kwashiorkor, Marasmus).
- Any non-compensated or uncontrolled condition, such as active tuberculosis, malignant disease, severe malaria, HIV, or other major infectious diseases
- Lactation, pregnancy (to be determined by adequate test) or inadequate contraception in females of childbearing potential for treatment period plus 2 months

Lack of suitability for the trial:

- Any history of prior anti-leishmania therapy
- Any condition which compromises ability to comply with the study procedures
- Concomitant serious infection other than cutaneous

Administrative reasons:

- Lack of ability or willingness to give informed consent (patient and/or parent / legal representative)
- Anticipated non-availability for study visits/procedures

3.2.3 Criteria for withdrawal

A premature withdrawal or discontinuance shall be construed as withdrawal procedures of the study after the first exposure to study treatment. Withdrawals before that time should be considered "failures of selection."

Patients may be withdrawn from the study at any time at its own request or on the basis of clinical trial investigator. However, patients who request the withdrawal will be strongly encouraged to conduct appropriate tests to make a valid assessment and possible inclusion in the analysis.

The reasons for withdrawal are classified as follows:

Lack of effectiveness: The patient showed inadequate response to treatment or deterioration.

Lack of tolerance: the dose-limiting intolerance reported by patients or observed by the doctor and found to be "probably" related to study treatment (and therefore does not refer to an aggravation of a preexisting condition).

An adverse event is considered dose limiting toxicity if he understands grade 3 or 4 of the common terminology criteria for adverse events (CTCAE) and is confirmed by a re-assessment within a week.

In the case of treatment of grade 3 transaminase elevation CTCAE, the investigator may decide to continue treatment until no manifestation of liver injury is observed (eg. Jaundice), additional control tests are designed to ensure patient safety.

The CTCAE grade 4 elevation of enzyme (s) impairment (s) or clinical signs of liver damage require immediate discontinuation of study treatment.

Intercurrent disease: A disease or condition that occurred or worsened during the study and prevented the completion of treatment and procedures of the study, however, no evidence of a causal relationship to study treatment, the adverse event record corresponding to classify causality as "unlikely" or "not assessable".

Withdrawal of consent / non-adherence: The patient, parent or legal guardian withdrew consent or the patient did not adhere to study procedures (must be verified if an adverse event caused withdrawal).

Other (s) reason (s): Reason for discontinuation not approached by any of the criteria above, p. ex., moving the patient, other reasons must be specified.

Patients who are withdrawn from the study will not be replaced.

3.3 Description of treatments

3.3.1 Study Medication

Description: Miltefosine is formulated as capsules in two dosages: 10 mg or 50 mg. These are packaged in blister packs containing 7 capsules; the blisters are packed in boxes of 8 units. The investigator or authorized person means a box for an eligible patient by writing the initials of the patient on the box label, ie, the capsules contained in a box are for use only a single patient, residual capsules can not be used by a another patient.

The batch number is documented in the certificate of analysis, to be held in the master file of the study, a copy will be included in *the investigator's file.*

Re-examination or expiration date: The medication must be used after re-examination or expiration date that is specified on the analysis certificate. The monitor of the study should commence appropriate measures in due time if the study medication is needed after that date. Should be made with written notice about the extent of the review / expiry date.

Responsibility at the center of the study: The researcher is responsible for proper storage and distribution of products in research and observe the relevant pharmaceutical regulations. The researcher must nominate a responsible person to care for the study medication.

Storage: The medication should be kept out of reach of children and protected from direct sunlight.

3.3.2 Appointment to the processing and recording

Patients who have been included in evaluations of selection for this research will be documented in an identification list + Patient Record Selection / enrollment.

Each eligible patient will be designated by consecutive numbers, starting at 1 for each age group. The number of patients will be documented in the List of ID + Patient Record Selection / enrollment.

Registration and randomization: the treatment will be allocated according to a randomization list generated by the biostatistics program.

Home Treatment: Treatment should be started up within 2 weeks after completion of evaluations of selection. Otherwise meeting the criteria for eligibility will be restored.

3.3.3 Administration of study treatment

Miltefosine

The dose and dosing schedule will be as follows:

Via:	Oral
Dose for Group 1:	Target dose of 2.5 mg / kg body weight per day;<br>daily dose rounded to the next increment of 10 mg;<br>Patients over 20 kg will receive 1 capsule of 50 mg per day
Dose for Group 2:	Patients> 25 kg body weight: 100 mg / day, or one capsule of 50 mg in the morning and one capsule of 50 mg at night and after meals<br><br>Patients <25 weight: 50 mg / day, ie a capsule of 50 mg in the morning after meal
Duration:	The duration of study treatment is 28 days

Standard treatment - Pentavalent Antimony (Glucantime ®)

Pentavalent antimony: with 5ml ampoules containing 81mg of pentavalent antimony (Sb^v) per ml:

Dose 20mg/Sb^v / kg / day administered intramuscularly or by intravenous injection (no need for dilution, application must be slow: last for about 5 minutes). It is necessary to rest after application.

Note: maximum daily dose for adults is 3 ampoules or 1.275mg Sb^v.

Modifications in the treatment

Reduce dose: A patient who does not tolerate the dose of Miltefosine used, will leave the treatment. No dose reduction is planned.

Discontinuation of study treatment: The criteria for withdrawal are presented in section 4.2.3.

Emergency measures

Adverse reactions known and associated with more intense dosing regimen proposed for Miltefosine are vomiting and diarrhea.

There must be an attentive observation and given the appropriate supportive treatment, adverse drug reactions suggested should be treated symptomatically.

Concomitant

All concomitant medications (including prophylactic medication, if any) and any treatments (prescribed and non-prescription) must be recorded in medical records, brand-name and generic name, route or formulation, dosing, indication and starting dates and end of the administration. Any changes that occur during the study should be documented, too.

To allow the symptomatic treatment of adverse effects and treatment of concomitant diseases not associated with cutaneous leishmaniasis, is decided by the investigator, is desirable.

Rescue treatment

Rescue treatment will be started if there is deterioration in clinical condition probably attributed to cutaneous leishmaniasis despite treatment of the study.

Rescue treatment for a patient who is not getting hit during or after the use of Miltefosine, will be the standard treatment, according to standard procedures of the institution of the researcher.

3.4 Evaluation of clinical response

Effectiveness

The eradication of Leishmania led to clinical cure is the goal of treatment.

A definite cure requires the complete healing of skin ulcerations at the end of 6 months of follow-up period.

Methods

Clinical evaluation, photographic documentation

Each ulcer is measured by size (two dimensional diameter, larger in mm), and evaluated for their degree of epithelialization. A standardized photograph will be taken before, during (14 days) at the end of treatment (28 days) and after treatment (1, 2, 6 months).

Parasitology

For each lesion that is not completely epithelialized, there will be a parasitologic selection (diagnosis). Parasitological examination of additional lesions that are not fully cured will be held 2 weeks after the end of treatment and / or during follow-up. Tissue samples will be collected by one of the following methods: skin smear, aspirate, biopsy.

Evidence of infection include:

Microscopic identification of amastigotes of leishmania in Giemsa staining direct (smear) and / or the demonstration of mobile promastigotes cultured aspirate and / or a biopsy of the lesion and / or by PCR identification.

The presence of parasites (the inclusion or treatment failure) is sufficiently demonstrated by a positive finding with one of three methods (swab / aspirate / biopsy) in a lesion.

Response criteria (efficacy)

Apparent cure

Complete healing of all ulcers until 2 months after completion of treatment.

Definitive cure

Complete re-epithelialization with a characteristic scar and no inflammationof all ulcers at the end of 6 months of follow-up period.

Partial healing

Incomplete epithelialization of one or more lesions.

Clinical failure

Any of the following points will qualify for treatment failure: residual lesions with the presence of parasites, or development of any new lesion or> 50% increase of lesions previously documented 2 months after the end of treatment or at any time during the monitoring period.

If a patient meets the criteria of partial healing, two months after the end of treatment, the response will be evaluated as clinical failure and the patient will receive salvage treatment.

If no parasitological analysis is performed 2 months after the end of treatment / or during the monitoring, evaluation of clinical failure will be based only on clinical criteria. A positive parasitological result at or after the 2 months after the end of treatment, always define a treatment failure.

Provision of patient

Apparent cure or partial cure: The patient will be monitored (up to 6 months after the end of treatment). Every patient who is not clinically cured 2 months after the end of treatment will receive rescue treatment.

Clinical failure at any time after the end of treatment: rescue treatment with parenteral standard Sb^v therapy.

Security - Adverse Events

Definitions

An adverse event (AE) is any untoward medical occurrence in a patient or individual participant in clinical research administered with a pharmaceutical product that does not necessarily have a causal relationship with this treatment. An adverse event (AE) can therefore be an unfavorable sign or unintentional including an abnormal laboratory finding (or vital, ECG, etc.), symptom or disease temporarily associated with the use of a drug under investigation, or related not this drug.

Serious adverse event (SAE) means any untoward medical occurrence that at any dose results in death, is a risk to the life, the patient requires hospitalization or prolongation of existing hospitalization, results in disability or significant disability or incapacity, is a congenital anomaly / inborn defect or other condition is a clinically important

A The term "life threatening" in the definition of "serious" refers to an event in which the patient is at risk of death at the time of the event, does not refer to an event that hypothetically might have caused the death would be more serious.

B clinically important conditions that may not result in death, be life-threatening or require hospitalization may be considered when SAE, based on appropriate clinical trial, may involve risk to the patient or require intervention to prevent one of the outcomes listed in the above definition .

Note: The term "severe" is often used to describe the intensity (severity) of an event (as in mild pain, moderate or severe), the event itself may be of relatively minor clinical significance (such as severe headache). Not the same as "serious", which is based on the outcome of event / patient or action criteria usually associated with events that pose a risk to the patient's life or vital function. Seriousness (not severity) serves as a guide for defining regulatory reporting obligations.

Procedures in case of adverse events

The investigator should ask the patient at each visit if he / she had any medical problem or not unusual.

All adverse events that occur during the study treatment should be documented, regardless of the assumption of a causal relationship in respective page of the report form (CRF). All symptoms / complaints that have occurred in the last 14 days before the first exposure to study treatment should be documented in "Complaints at Baseline 'medical records.

The documentation of adverse events including start and end date, severity, according to the CTCAE criteria, and seriousness. The investigator should also assess the likelihood of a causal relation of adverse event to study medication as probable, not assessable, or unlikely. The information provided in the investigator's brochure can give support to this evaluation.

Documentation of security parameters for routine

A value outside the normal range or reference in a routine safety assessment, such as laboratory tests, vital signs, ECG, can mean an adverse finding. If the investigator finds that the abnormality has a major relevance, it must also register it in Adverse Event case report form. If the findings contribute to a clinical diagnosis (eg., Hepatitis, in the case of elevated liver enzymes), then this diagnosis should be recorded as an adverse event.

An abnormality should be considered an important clinical relevance if:

represent a serious adverse event, or led to premature discontinuation of the study, or required a therapeutic measure.

With respect to laboratory safety parameters, the investigator must classify each value outside the normal range at:

1. Abnormal but of secondary importance.

2. Abnormal and important relevance (seriously, leading to discontinuation, required a therapeutic measure) should be made to their record in the clinical record.

3. Invalid value, must be specified the reason for this trial.

Documentation of adverse reactions expected

The following selected adverse reactions (= causality adverse events with "probable" with the study drug Miltefosine) will not be recorded in the Adverse Event report form, but separate modules will be documented in medical records:

Vomiting: documentation of intensity (according to the scale CTCAE)

Diarrhea: documentation of intensity (according to the scale CTCAE)

Safety parameters

The following safety assessments should be performed routinely with every patient. For more details on the frequency and extent, see Flowchart (item 4.5).

Medical laboratory tests

Clinical laboratory tests comprise the following parameters:

Hematology	leukocytes, hemoglobin, platelets (thrombocytes)
Electrolytes	sodium, potassium
Substrates	Albumin levels, total bilirubin, creatinine, urea
Enzymes	SGOT / AST SGPT / ALT, alkaline phosphatase
Urinalysis	record of blood and protein only
Pregnancy	β–HCG, presence or absence, in women who may become pregnant

Additional tests will be performed when clinically indicated.

Criteria and definitions

Normal levels of local laboratory of the participating institution.

The investigator will classify each laboratory value outside the normal range:

1. Abnormal but of secondary importance

2. Abnormal and important relevance (seriously, led to discontinuation, requested a therapeutic measure) should be made to their record in the clinical record

3. Invalid value, must be specified the reason for this trial.

Vital parameters

Vital parameters to be recorded include systolic blood pressure, diastolic blood pressure, pulse, body weight and body temperature.

The investigator should comment on the respective section of medical records, the changes compared to baseline that are considered clinically relevant.

4. STUDY PROCEDURES

4.1 Pre-study evaluation / selection

Before the first administration of study medication, the investigator will inform the patient verbally and in writing about the potential benefits and any risks associated with their participation in this study and obtain a consent in writing.

The assessment team to verify compliance with the criteria for inclusion / exclusion include:

**Medical history:**	General medical history (demographic data, sensitivity to medications and allergies, medical history and surgical significance), concomitant medication,<br>history of leishmaniasis, previous treatments
**Physical examination:**	Will be recorded: blood pressure, pulse, weight, height, temperature.
**Medical laboratory tests ***	CBC (no differential), platelets, stool examination<br>HIV serology by ELISA or rapid test<br>Biochemistry (Na^{+}, K^{+}), urea, creatinine, ALT, AST, LDH, alkaline phosphatase<br>Urinalysis (record of blood and protein only)<br>Pregnancy test: β–HCG serum<br>Hepatitis C antibodies, HBsAg, anti HBc

*) All laboratory studies conducted in laboratory validated and reviewed. Abnormal values (values outside the normal laboratory values - 3 benchmarks) will be specifically addressed in patient records.

A second evaluation (at baseline) to verify adherence to the criteria for inclusion / exclusion will be necessary if the time between the assessment team and the first administration of study medication more than two weeks. Include the variables as above for the assessment of selection. In a female patient that requires a waiting period for repayment of a pregnancy test, to repeat the screening tests is unnecessary in the absence of clinical deterioration.

4.2 Assessments during the study

Safety assessments:

Vital parameters	7, 14, 21
Adverse Events	continuously
Laboratory	
- CBC	7, 14, 21
- Clinical Biochemistry	7, 14, 21

4.3 Assessments at the end of treatment

The end of treatment will be the 28th day in the case of regular treatment. In case of premature discontinuation, all assessments listed below will be executed whenever the treatment is discontinued.

Variable security:

Vital parameters

Adverse Events

Laboratory tests (CBC, clinical biochemistry, urine)

Pregnancy test in women who may become pregnant

Overall assessment: Reason for discontinuation, overall assessment of tolerability
A variation of the assessments plus / minus 2 days is acceptable. If these variations are not met, the reason should be documented in the appropriate page of medical records.

4.4 Assessments 2 weeks, 1 month and 2 months after the end of the treatment

Efficacy variables: Lesion size / epithelization grade / size of the infiltration in the lesion
Photograph of each lesion
Leishmania parasitology
Response to study treatment

4.5 Assessments 6 months after the end of the treatment

Efficacy variables: Lesion size / epithelization grade / size of the infiltration in the lesion
Photograph of each lesion
Leishmania parasitology

Variable security: Vital parameters
Laboratory parameters (CBC, clinical biochemistry)

Overall Assessment Results of the study and response to therapy

4.6 Flowchart

	Visit	1 [a)]	2	3	4	5	6 [b)]	7	8	9	10	11
Evaluation	Day	Before therapy	Day 1	Day 7	Day 14	Day 21	Day 28 or final therapy	2 weeks after the end of therapy	1 month after the end of therapy	2 months after the end of therapy	4 months after the end of therapy	6 months after the end of therapy
Informed consent		x										
Medical history		x										
History leishmaniasis		x										
Parasitology (fecal / blood)		x										
Pregnancy test (women of childbearing age)		x [c)]			X							
HIV Testing		x										
Effectiveness												
- Parasitology		x					x	x	x	x	x	x
- Size / degree of epithelization / area of infiltration		x		x	x	x	x	x	x	x	x	x
- Photo [d)]		x			x		x	x	x	x		x
Laboratory:												
- CBC		x		x	x	x	x		x			
- Biochemistry		x		x	x	x	x		x			
- Urine type 1		x					x		x			
- ECG (control patients)		x					x					
Vital signs		x	x	x	x	x	x		x	x	x	x

a Within the 14 days before the 1st dose (except for parasitological examination of lesions, which will be valid within the last 4 weeks)
b It should be evaluated fully when treatment is discontinued. The reason (s) for discontinuation is (are) documented (s). This applies also to patients who withdrew from the study due to any reason.
c Evaluation of selection performed in the first half of the menstrual cycle (<10 days of the cycle) in the case of a negative pregnancy test before randomization, the treatment can be initiated without waiting period.

Evaluation of selection performed in the second half of the menstrual cycle (> 10 days) pregnancy test followed by a waiting period of two weeks, during which they should be used adequate contraception. After the waiting period should be performed a confirmatory test of pregnancy, before randomization.
d It should be taken a standard photo (in accordance with the procedure approved by the center) of each lesion.

5. LEGAL ASPECTS OF BIOETHICS, BIOSAFETY, INTELLECTUAL PROPERTY AND OTHER LEGAL DETERMINATION

5.1 Ethical general aspects

The study will be conducted in accordance with the following guidelines:
• Declaration of Helsinki (Edinburgh, 2000), copy the file researcher
• Note for Guidance on Good Clinical Practice - ICH (CPMP/ICH/135 = 95), the file copy of the investigator
• Resolution 196/96, 251/97 and 292/99 of the CNS and subsequent dealing with Standards and Guidelines for Research Involving Human Subjects.

5.2 Consideration of risk-benefit

Cutaneous leishmaniasis is a disease that is associated with clinically relevant morbidity, especially if the infection is widespread, diffuse or involve children (20% of cases), evolve with secondary infection or present a difficult approach.
The current standard treatment is done with pentavalent antimonials. These drugs are parenteral use of exclusive and their use is associated with sometimes severe toxicity, including pancreatitis and cardiac arrhythmias. The restrictions also apply to the use of amphotericin B, a drug of second choice in the treatment of disease that is associated with the occurrence of medication-induced fever and renal dysfunction. Amphotericin B requires hospitalization with elevation in the final cost of treatment.
The miltefosine was safe and effective in treating patients with LC (and LV). Its main adverse effects are related to the gastrointestinal tract, the liver and renal function and rarely dose-limiting. The medication has the added advantage of oral administration.
Preclinical studies indicated a teratogenic potential of Miltefosine, but no clinical experience is available on the use of this medicine during pregnancy in humans. Therefore, pregnancy should be strictly avoided during this study and in a period of 2 months after treatment ends. Preclinical studies in rats showed a dose effect and time-dependent in male fertility, no corresponding toxicity was observed in dogs. The after-treatment of Indian patients males during the reproductive performance and semen analysis before and after the use of Miltefosine (Colombia) as well as after treatment only (in India) did not suggest an adverse effect of Miltefosine on the male fertility.

5.3 Patients protection

Before the start of the research, the study protocol will be reviewed and forwarded for approval by the ethics committee in research (CEP/IRB) and National Committee for Ethics in Research (CONEP). The patient will be informed verbally and in writing on the nature of the study, the risks and benefits, the discomfort to which the patients will be exposed and also its right to discontinue your participation at any time in its sole discretion. The content of the Informed Consent Form Informed Consent / patient information in writing (archive researcher) will be explained. Patients will be asked to give their consent (in writing or by fingerprint) before inclusion. In the case of an illiterate patient, the information session will be attended by an independent witness to ensure that the content of written information to patient / term of informed consent has been explained to the patient. The witness also sign (in writing or thumbprint) consent.
For younger patients, a parent or legal representative attend the information session and the parent / legal representative and (if able) the patient shall consent (in writing or fingerprint) before inclusion. The term of consent will be reviewed and approved by the CEP / IRB.

The patient information and consent form will include the following statement on the treatment and compensation for damages: if you were to suffer some damage as a result of their participation in this study, available treatment, as described in section V.6 of resolution 196 / 96 of the National Health Council, Ministry of Health of Brazil, which states, "The research subjects who suffered from any kind of damage is foreseen or not the consent form and a result of their participation, besides the right to comprehensive care entitled to compensation. "The cost of this treatment will be borne by the sponsor. You will be closely watched by doctors in the study during the search. If you present any serious reaction to the study drug, you will be treated immediately. You will be informed about any new information learned during the study that could change his will to remain in it.

6. REFERENCES

1. Berman JD. Human Leishmaniasis: Clinical, diagnostic, and chemotherapeutic developments in the last 10 years. Clin Infect Dis 1997; 24:684-703
2. Burk K, David M, Junge K, Sindermann H. Overview on the clinical development of Miltefosine solution (Miltex®) for the treatment of cutaneous breast cancer. Drugs of Today 1994; 30 (Suppl. B):59-72
3. Croft SL, Snowdon D, Yardley V. The activities of four anticancer alkylphospholipids against Leishmania donovani, Trypanosoma cruzi and Trypanosoma brucei. J Antimicrob. Chemoth. 1996; 38:1041-1047
4. Escobar P, Yardley V, Croft SL. Activities of hexadecylphosphocholine (Miltefosine), AmBisome, and sodium stibogluconate (Pentostam) against Leishmania donovani in immunodeficient scid mice. Antimicrobial Agents and Chemotherapy, 2001; 45: 1872-5
5. Herwaldt BL, Berman JD. Recommendations for treating leishmaniasis with sodium stibogluconate (Pentostam) and review of pertinent clinical studies. Am J Trop Med Hyg. 1992; 46:296-306
6. Hilgard P, Klenner T, Stekar J, Unger C. Alkylphosphocholines: a new class of membrane-active anticancer agents. Cancer Chemother. Pharmacol. 1993; 32:90-5
7. Jha TK, Sundar S, Thakur CP, Bachmann P, Karbwang J, Fischer C, Voss A, Berman J. Miltefosine, an oral agent, for the treatment of Indian visceral leishmaniasis. New Eng J Med 1999;341:1795-1800
8. Pronk LC, Planting ASTh, Oosterom, R, Drogendijk TE, Stoter G., Verweij J. Increases in leucocyte and platelet counts induced by the alkyl phospholipid hexadecylphosphocholine. Eur J Cancer 1994; 30A:1019-22
9. Soto J, Toledo J, Gutierrez P, Nicholls RS, Padilla J, Engel J, Fischer C, Voss A, Berman J. Treatment of American cutaneous leishmaniasis with Miltefosine, an oral agent. Clinical Infectious Diseases 2001; 33:e57-61
10. Soto J, Arana BA, Toledo J, Rizzo N, Vega JC, Diaz A, Luz M, Gutierrez P, Arboleda M, Berman JD, Junge K, Engel J, Sindermann H. Miltefosine for New World cutaneous leishmaniasis: placebo-controlled multicenter study. Clinical Infectious Diseases 2004, Clin Infect Dis. 2004;38(9):1266-72.
11. Sundar S, Gupta LB, Makharia MK, Singh MK, Voss A, Rosenkaimer F, Engel J, Murray HW. Oral treatment of visceral leishmaniasis with Miltefosine. Ann Trop Med & Parasitology 1999;93:589-97
12. Sundar S, Makharia A, More DK, Agraval G, Voss A, Fischer C, Bachmann P, Murray HW. Short course of oral Miltefosine for treatment of visceral leishmaniasis. Clinical Infectious Diseases 2001; 31:1110-3
13. Sundar S, Rosenkaimer F, Makharia MK, Goyal AK, Mandal AK, Voss A, Hilgard P, Murray HW. Trial of oral Miltefosine for visceral leishmaniasis. The Lancet 1998;352:1821-3
14. Verweij J, Plating A, van der Berg M, Stoter G. A dose finding study of Miltefosine (hexadecylphosphocholine) in patients with metastatic solid tumors. J Cancer Res Clin Oncol, 1992; 118:606-8

15. Werbowetz KA. Promising therapeutic targets for antileishmanial drugs. Expert Opin Ther Targets 2002; 6: 407-22
16. WHO: The Leishmaniasis: Report of a WHO Expert Committee. WHO Tech.Rep.Ser. 701:99, 1984
17. WHO. Leishmania/HIV co-infection in South-western Europe 1990-1998 – Retrospective Analysis of 965 cases. WHO Report: WHO/LEISH/2000.42
18. WHO/TDR. Special feature: Miltefosine
(i) Ganguly NK. Oral Miltefosine may revolutionize treatment of visceral leishmaniasis (p. 2)
(ii) Ridley RR and Karbwang J. Miltefosine discovery and development and the TDR product development paradigm (p. 2-3)
(iii) Engel J. Miltefosine, the story of a successful partnership. disease endemic country – TDRtgphar888maceutical industry (Zentaris) (p. 5)
juhhTDR News 2002; No. 68
19. Manual de Controle da Leishmaniose Tegumentar Americana. Ministério da Saúde. Fundação Nacional de Saúde. 5 edição revisada, 2000.
